# The Influence of Medical Studies upon Religious Belief

**Published:** 1875-09

**Authors:** Theodore Dimon

**Affiliations:** Auburn, N. Y.


ART. III.—*The Influence of Medical Studies upon Religious Belief*. Read betore the Cayuga County Medical Society. By Theodore Dimon, M. D., of Auburn, N. Y.

[Published by vote of the Society.]

The modern study of science tends towards infidelity in religion, and the profession of medicine is especially open to this charge. Dealing with the human body as a machine that we are called upon to study as a material existence, and to regulate its operations by material means, we are led to view all its conditions and manifestations as material results of organization, and to deny the existence of a soul, or of any separate individuality, having character, responsibility, or fate, unconnected with the body. Called upon, as our profession also is more than any other, to study the natural sciences and to employ in our investigations the mathematics and natural philosophy, and in our practice to rely upon fixed laws and the obtaining of material results, that we may prevent as long as possible our fellow beings from leaving this world's life, there is this additional impulse towards materialism among us. The question arises whether such study and practice need to or should have this effect with us, and whether scientific pursuits generally should affect any of their followers in this way.

I use the term infidelity in religion only in its strict sense of want of faith. All religions call for faith or belief in something not capable of demonstration or proof, and some of them for faith in things which are capable of being proved to be absurd and untrue. In this point of view their very foundation support rests on a basis so different from that upon which all science rests, as to raise a hostile disposition between their respective followers when brought in collision. Save in one redeeming feature which runs through them all, all other religions save the Christian may be left to one side in treating this question. That one feature is the belief in a creating, governing being, or set of beings, to whom mankind is responsible.

I affirm that the tendency of the study of physical science ought to be to establish a belief in the existence of a creative power. Whether, having created, such power also governs, and if mankind is responsible to it for his conduct, are the further points upon which science and religion begin to diverge. That they should do so or need to do so, whether on the contrary they do not, each demand the other to make out any reasonable scheme of existence for creation or creating power or for God and His works, is the subject proposed to be considered in this paper.

In discussing it, it seems proper to say that we should avoid some positions which it is thought belong to the advocates on either side rather than to the subject itself. One of the most prominent of these is the making a science of Theology, when there can be confessedly no science, and indulging in the absurdity of appealing to reason in its support, and at the same time condemning all human reasoning on divine subjects as impious and sinful. The foundation of theology as a science was laid according to the Bible, at the very beginning of human existence, in the Garden of Eden, Satan being its professor and Eve the student, and the clear and unmistakable foundation of God's religion was then and there laid, in simple faith and obedience, as contra-distinguished from theologic science. And the evil and fatal origin of theologic science has given character to its subsequent history, in all the wars, tortures and martyrdoms, in all the pride, vain glory and deceit it has produced. "And the Lord said unto Moses: make thee a fiery serpent and set it on a pole; and it shall come to pass that every one who is bitten when he looketh upon it shall live. And Moses made a serpent of brass and put it upon a pole." Now suppose that the Israelites had fallen to disputing and to torturing and burning one another at the stake as to whether that serpent on the pole simply consisted of brass, or was actually a living serpent, instead of looking to it in simple faith that it would save them; the story would then have furnished a good illustration of the distinction between theology and religion. Witnesss the history of the dogma of the real presence. A prominent position on the other side, taken by scientists, is one which seems equally untenable, and that is to require moral questions to stand on the same basis as physical questions, and to apply the rule *falsus in uno falsus in omnibus* to the former. The physicist cries out against revelation when he discovers a falsity in some purported statement of revelation, false in one thing false in all, while he knows and claims that to discover one error or falsity in science is but a step forward in the road of truth, and that *exceptio probat regulum.*

I have said that the tendency of the study of physical science ought to be to establish a belief in the existence of a creative power. It is not proposed to go into the proofs and evidences on this point, because it must be taken by all reasoning beings as self-evident. A thing made requires a maker. It did not make itself.

> "What but God!
> Inspiring God! who boundless spirit all,
> And unremitting energy, pervades,
> Adjusts, sustains, and agitates the whole.
> He ceaseless works alone; and yet alone
> Seems not to work; with such perfection framed
> In this complex, stupendous frame of things.
> But though concealed, to every purer eye
> The informing author in his works appears."

But you ask who made the maker? The human mind *must* stop there. And because it must stop there, that must be received as an axiom. Physical science takes account only of things made, and of their actions and reactions on each other, so far as our faculties and appliances will enable us to ascertain them. It is constantly studying laws and causes in nature, and as constantly, from the very nature of the human mind, it requires a first cause and law-giver. And the further the wonderful developments are followed, as in Astronomy, into boundles space, till the throbbing brain is ready to burst in the vain endeavor to grasp the whole, the more wonderful becomes the maker.

The contemplation of the designs, the beauties, the bounties of creation evince an intelligent and benificent creator, and not a mere first cause; and here both science and religion may start in accord in asking the question, why have these things been created? And here to both comes, in the words of Castellar, "the mysterious idea of a personal God, from which descends inspiration upon the arts, light upon the sciences, the hope of immortality upon this short and fragile life." Science must, by the very necessity of the case, leave this question to be answered by religion, or it must remain without any answer at all. Science requires, however, the question to be put, because it has been all the while contemplating design in all the physical steps it has traced. An intelligent and benificent creator being conceded, and the mere scientist, being obliged to stop here with his question of the why, is landed in a friendly and not hostile attitude by the side of the religionist, whom a necessity arising just as naturally and imperatively in his contemplations of human character, responsibility and destiny requires to ask the same question. Physics are a revelation to our senses; religion must be a revelation to the mind or soul, and on that account, of the character called miraculous. The basis of the one is fact, of the other faith; for on this latter basis alone can this momentous question be answered. Every part and parcel of physical science, every created, every existing thing, put these imperative questions to man. Why, so far as man is concerned, has all this been done? Why does he exist? What is his relation and duty to his creator? Mankind has endeavored to answer them of himself in vain. No process of reasoning, no mental or moral philosophy, no system of theology ever has or ever will answer them. All such efforts have been easily and successfully overthrown, leaving but blanks and vacuums, but I don't knows, between man and God. As said before in the history of all religions, all the truth and revelation there has been in them, and some of this has been in them all, has been faith in an intelligent and beneficent God, to whom man is accountable for his conduct, so far redeeming them all from the superstitions and errors, with which the devices and desires of our wayward nature has surrounded them. In physical science God has made a direct revelation to our physical senses; in morals has he left us without a revelation so imperatively necessary? To so believe, is to leave man, with character and accountability, floating in a sea of disappointments and uncertainties which would end in the insanity of atheism. A revelation, then, divine, direct, miraculous if you please, is it not then, not only a necessity, but a logical expectation —a revelation as much demanded by physical science as by man's moral and accountable nature. This revelation, as above stated, has so far as really made, been accorded to man always, and has been all the truth and reality and redeeming characteristics of all the religions man has had. God has never left man without a witness of himself. In the Bible, in the Christian religion, the dearest, the most direct, the most unmistakable, the only entirely divine revelation of true religion has been made. Let skepticism and science weed out of the Bible as much of error and absurdity as you please, there are none of them religion. The Bible does not reveal physical science, nor is it a history of events or processes; and no matter whether the weak and vain human agents who received and kept this revelation have surrounded it with their own added and unnecessary and obscuring statements and superstitions, as we know from its own testimony they were constantly doing, yet ever and ever the real divine revelation shines out only the brighter and clearer amidst all these. In Eden the simple faith and obedience due from man to God, and not the physical science of geology, was the revelation. But it is unaccountable, nevertheless, that anybody can read the sublime story of the creation, in which there is no revelation at all, and only find in it an occasion for criticizing the six days and six nights. All the way through the Old Testament and the New there is a plain clear stream of revelation of God's will to man, and of man's present and future relations to him, while the accompanying history is made up of man's perversion of the spirit and design of the revelation, and of his constantly leaving the duty of simple faith and obedience for theological systems and plans and devices, and these so plainly at variance with the divine will were then, as now, made the grounds of attack against the authenticity of the revelation itself, while in fact they only the more show the necessity and make evident the proof of the true religion there revealed. I do not propose to preach a sermon, but rather to show that scientific pursuits, and particularly the study of our profession, should not have a tendency to induce skepticism on the subject of the Christian religion; and I will only add that its practice should have the very contrary effect. For how can we stand at the bedside of a dying human being and believe for a moment that that being, now existing with intellect to range the earth and the heavens, with emotions human and divine, with character and conscience, is in a few hours or minutes to become a mere disgusting mass of dead matter, and that the end?

